# Valproic acid inhibits adhesion of vincristine- and cisplatin-resistant neuroblastoma tumour cells to endothelium

**DOI:** 10.1038/sj.bjc.6603777

**Published:** 2007-05-15

**Authors:** R A Blaheta, M Michaelis, I Natsheh, C Hasenberg, E Weich, B Relja, D Jonas, H W Doerr, J Cinatl

**Affiliations:** 1 Zentrum der Chirurgie, Klinik für Urologie und Kinderurologie, Johann Wolfgang Goethe-Universität, Frankfurt am Main, Germany; 2 Zentrum der Hygiene, Institut für Medizinische Virologie, Johann Wolfgang Goethe-Universität, 60590 Frankfurt am Main, Germany

**Keywords:** neuroblastoma, chemoresistance, valproic acid, adhesion, NCAM

## Abstract

Drug resistance to chemotherapy is often associated with increased malignancy in neuroblastoma (NB). In pursuit of alternative treatments for chemoresistant tumour cells, we tested the response of multidrug-resistant SKNSH and of vincristine (VCR)-, doxorubicin (DOX)-, or cisplatin (CDDP)-resistant UKF-NB-2, UKF-NB-3 or UKF-NB-6 NB tumour cell lines to valproic acid (VPA), a differentiation inducer currently in clinical trials. Drug resistance caused elevated NB adhesion (UKF-NB-2^VCR^, UKF-NB-2^DOX^, UKF-NB-2^CDDP^, UKF-NB-3^VCR^, UKF-NB-3^CDDP^, UKF-NB-6^VCR^, UKF-NB-6^CDDP^) to an endothelial cell monolayer, accompanied by downregulation of the adhesion receptor neural cell adhesion molecule (NCAM). Based on the UKF-NB-3 model, N-myc proteins were enhanced in UKF-NB-3^VCR^ and UKF-NB-3^CDDP^, compared to the drug naïve controls. p73 was diminished, whereas the p73 isoform deltaNp73 was upregulated in UKF-NB-3^VCR^ and UKF-NB-3^CDDP^. Valproic acid blocked adhesion of UKF-NB-3^VCR^ and UKF-NB-3^CDDP^, but not of UKF-NB-3^DOX^, and induced the upregulation of NCAM surface expression, NCAM protein content and NCAM coding mRNA. Valproic acid diminished N-myc and enhanced p73 protein level, coupled with downregulation of deltaNp73 in UKF-NB-3^VCR^ and UKF-NB-3^CDDP^. Valproic acid also reverted enhanced adhesion properties of drug-resistant UKF-NB-2, UKF-NB-6 and SKNSH cells, and therefore may provide an alternative approach to the treatment of drug-resistant NB by blocking invasive processes.

Multiple-agent chemotherapy is the conventional therapy for patients with advanced stages of neuroblastoma (NB) and disseminated NB. However, drug resistance arises in the majority of stage IV and relapsed NB, often leading to treatment failure (Keshelava *et al*, 1998). Development of novel antitumoural strategies is therefore highly desired to overcome resistance mechanisms and to prevent tumour progression. Molecules modulating cellular function have been identified in the majority of tumours and their manipulation might be the key to decreasing malignancy.

Histone deacetylases (HDAC) represent one of the most important intracellular targets, as these molecules modulate a wide variety of cellular functions. Abnormal histone acetylation status can result in undesirable phenotypic changes, including developmental disorders and cancer. Indeed, aberrant histone acetylation may be an aetiological factor in several types of cancer by derepressing gene transcription. Hence, HDAC inhibitors may be useful for cancer prevention, due to their ability to ‘reactivate’ the expression of epigenetically silenced genes, including those involved in differentiation, invasion and metastasis. Most notably, recent data indicate that HDAC inhibition may be successful in treating refractory or relapsing tumours after conventional chemotherapy. Histone deacetylase inhibition has been demonstrated to block cell growth of drug-resistant small-cell lung cancer lines (Tsurutani *et al*, 2003), abrogate resistance in breast cancer cells (Hirokawa *et al*, 2005) and induce apoptosis in drug-resistant ovarian cancer cells (Sonnemann *et al*, 2006), myeloma cells (Maiso *et al*, 2006) and hepatoma cell lines (Pathil *et al*, 2006).

The branched-chain fatty acid valproic acid (VPA) has been shown to possess HDAC inhibitory properties and to affect the growth and survival of tumour cells *in vitro* and *in vivo* (Cinatl *et al*, 1996; Blaheta *et al*, 2005). This is highly relevant since VPA is an established drug in the long-term therapy of epilepsy. It can be applied orally, negative side effects are rare and it demonstrates expedient pharmacokinetic properties. To clearly assess whether VPA might be of benefit in treating relapsed NB, we evaluated the potential of therapeutic VPA concentration to block the interaction of drug-resistant NB cells with vascular endothelium. The experiments were based on an *in vitro* model of acquired drug resistance (Kotchetkov *et al*, 2003, 2005), closely resembling progressive NB disease through long-term treatment of NB cell lines with vincristine (VCR), cisplatin (CDDP) or doxorubicin (DOX) to establish the resistant tumour cell sublines UKF-NB-3^VCR^, UKF-NB-3^CDDP^ and UKF-NB-3^DOX^. A co-culture binding assay allowed the analysis of NB cells that adhered to an endothelial cell monolayer. Since surface receptors are strongly involved in tumour invasion and data have indicated that changes in neural cell adhesion molecule (NCAM, CD56) expression play an essential part in the progression of NB, we investigated NCAM expression (Blaheta *et al*, 2002), and the NCAM regulating proteins N-myc, p73 and deltaNp73 (Blaheta *et al*, 2004) under the influence of VPA.

## MATERIALS AND METHODS

### Cell cultures and induction of drug resistance

The N-myc amplified human NB cell lines UKF-NB-2, UKF-NB-3 and UKF-NB-6 were established in our laboratory from bone marrow metastases. The CDDP-, VCR- and DOX-resistant UKF-NB-2, UKF-NB-3 and UKF-NB-6 sublines were established by exposing the parental cells to increasing concentrations of the drugs. Solutions of CDDP (Gry-Pharma, Kirchzarten, Germany), VCR (Sigma-Aldrich, Deisenhofen, Germany) and DOX (Farmitalia, Milan, Italy) were prepared in accordance to the manufacturer's instructions. The initial CDDP, VCR and DOX concentrations were 50, 0.2 and 2 ng ml^−1^ medium, respectively. The resistant sublines were grown for more than 6 months in Iscove's modified Dulbecco's medium (Gibco, Karlsruhe, Germany) supplemented with 10% fetal calf serum (FCS, Gibco) and either 10 ng ml^−1^ VCR (UKF-NB-2^VCR^, UKF-NB-3^VCR^, UKF-NB-6^VCR^), 20 ng ml^−1^ DOX (UKF-NB-2^DOX^, UKF-NB-3^DOX^, UKF-NB-6^DOX^), or 1000 ng ml^−1^ CDDP (UKF-NB-2^CDDP^, UKF-NB-3^CDDP^, UKF-NB-6^CDDP^). The clinical relevance of drug-resistant UKF-NB-2 and UKF-NB-3 sublines has been examined before in a xenograft setting (Kotchetkov *et al*, 2003, 2005). Multidrug resistant, not N-myc amplified SKNSH, were derived from LGC Promochem, Wesel, Germany. Cells were subcultured at 5-day intervals.

Chemoresistance of the indicated cell lines was verified by the MTT test (Kotchetkov *et al*, 2003). The resistant cell lines showed at least 20-fold increase in resistance to the drugs, expressed as IC_50_.

Human endothelial cells (HUVEC) were isolated from human umbilical veins and harvested by enzymatic treatment with chymotrypsin. Human endothelial cells were grown in Medium 199 (M199; Biozol, Munich, Germany), supplemented with 10% FCS, 10% pooled human serum, 20 *μ*g ml^−1^ endothelial cell growth factor (Boehringer, Mannheim, Germany), 0.1% heparin, 100 ng ml^−1^ gentamycin and 20 mM HEPES buffer (pH 7.4). Subcultures from passages 2–6 were selected for experimental use.

### VPA treatment

Parental NB tumour cells and their drug-resistant sublines were treated with VPA (gift from GL Pharma GmbH, Lannach, Austria) at a final concentration of 1 mM for 3 or 5 days. Tumour cell adhesion, NCAM, p73, deltaNp73 and N-myc expression were then measured in VPA-treated cells. Results were compared to untreated controls. Viability of tumour cells in presence of VPA was assessed by propidium iodide dsDNA intercalation.

### Tumour cell adhesion

Human endothelial cells were transferred to six-well multiplates (Falcon Primaria; Becton Dickinson, Heidelberg, Germany) in complete HUVEC medium. When confluency was reached, 0.5 × 10^6^ parental NB tumour cells or their drug-resistant sublines (VPA treated *vs* non-treated) were carefully added to the HUVEC monolayer for 60 min. Subsequently, non-adherent tumour cells were washed off using warmed (37°C) M199. The adherent cells were fixed with 1% glutaraldehyde and counted in five different fields (5 × 0.25 mm^2^) using a phase contrast microscope (20 × objective) to calculate the mean cellular adhesion rate.

### Cell proliferation

Proliferative activity of NB tumour cells and HUVEC was estimated by the PicoGreen assay as described elsewhere (Blaheta *et al*, 1998). Briefly, at several time points after plating the cells in six-well multiplates, culture medium was removed and cells were digested with papain (0.125 mg protein ml^−1^) for 20 h at 60°C. Thereafter, the fluorescent dye PicoGreen (MoBiTec, Goettingen, Germany), which shows high specificity for dsDNA, was added (1 : 200 dilution) for 10 min at 20°C. Fluorescence intensity was determined using a computer-controlled fluorescence reader (Cytofluor 2300 plate scanner; Millipore, Eschborn, Germany) at *λ*ex=485 nm and *λ*em=530 nm.

### Evaluation of NCAM surface expression

Neuroblastoma cells were disaggregated mechanically, washed in blocking solution (PBS, 0.5% BSA) and then incubated for 60 min at 4°C with an FITC-conjugated monoclonal antibody anti-CD56 which detects the NCAM 120, 140 and 180 kDa isoform (clone 16.2). Neural cell adhesion molecule expression of NB cells was then measured using a FACscan (Becton Dickinson; FL-1 H (log) channel histogram analysis; 1 × 10^4^ cells/scan) and expressed as mean fluorescence units (MFU). A mouse IgG1-FITC was used as the isotype control.

To explore NCAM localisation, tumour cells were transferred to round cover slips, which were placed in a 24-well multiplate. Upon reaching confluency, cell cultures were washed two times with PBS (with Ca^2+^ and Mg^2+^) and then fixed in cold (−20°C) methanol/acetone (60/40 v/v). Subsequently, cells were washed again with PBS (without Ca^2+^ and Mg^2+^), and later once with blocking buffer (0.5% BSA in PBS without Ca^2+^ and Mg^2+^). After removing the washing buffer, cells were incubated for 60 min with FITC-conjugated anti-NCAM monoclonal antibody. To prevent photobleaching of the fluorescent dye, cover glasses with stained cells were taken out of the wells and the residual liquid was removed. The cells were then embedded in an antifade reagent/mounting medium mixture (ProLong™ Antifade Kit, MoBiTec) and mounted on slides. The slides were viewed using a confocal laser-scanning microscope (LSM 10; Zeiss, Jena, Germany) with a plan-neofluar × 100/1.3 oil immersion objective.

### Western blot analysis

Total NCAM content in NB cells was evaluated by Western blot analysis: tumour cell lysates were applied to a 7% polyacrylamide gel and electrophoresed for 90 min at 60 V. The protein was then transferred to nitrocellulose membranes. After blocking, the membranes were incubated overnight with the anti-NCAM antibody (dilution 1 : 1000). HRP-conjugated goat anti-mouse IgG (Upstate Biotechnology, Lake Placid, NY, USA; dilution 1 : 5000) served as the secondary antibody. The membrane was briefly incubated with ECL detection reagent (ECL™, Amersham/GE Healthcare, München, Germany) to visualise the proteins and exposed to an x-ray film (Hyperfilm™ EC™, Amersham).

### Semi-quantitative reverse transcription/polymerase chain reaction

Total RNA was extracted and purified with Trizol reagent according to the manufacturer's instructions and treated with RNase-free DNase. The NCAM primer sequences were as follows: for NCAM-180: 5′CGAGGCTGCCTCCGTCAGCACC 3′ and 5′CCGGATCCATCATGCTTTGCTCTCG 3′; for NCAM-140: 5′GAACCTGATCAAGCAGGATGACGG 3′ and 5′CCGGATCCATCATGCTTTGCTCTCG 3′ (Kleinschmidt-DeMasters *et al*, 1999). Internal controls for the reverse transcription/polymerase chain reaction (RT-PCR) reaction was performed by running parallel reaction mixtures with the housekeeping gene GAPDH: 5′ATCTTCCAGGAGCGAGATCC 3′ and 5′ACCACTGACACGTTGGCAGT 3′. Ribonucleic acid (1–10 *μ*g) was reverse transcribed and the resulting cDNA directly added to the PCR. Amplification reactions (20 *μ*l) were performed in the presence of 1/10 (2 *μ*l) of the cDNA reaction, with an initial incubation step at 94°C for 1 min. Cycling conditions consisted of denaturation at 94°C for 1 min, annealing at 55°C for 1 min and extension at 72°C for 1 min over a total of 30 cycles. The reaction was completed by another incubation step at 72°C for 10 min. The PCR products were subjected to electrophoresis in 2% agarose gel and visualised by ethidium bromide.

### Evaluation of p73, deltaNp73 and N-myc

N-myc, p73 and deltaNp73 were evaluated by flow cytometry. To allow intracellular staining, tumour cells were fixed and permeabilised by methanol–acetone (1 : 1, −20°C) before the antibodies were added. Monoclonal anti-N-myc antibody was from Calbiochem (clone NCM II 100; mouse IgG1; Calbiochem, Bad Soden, Germany). p73 was measured using monoclonal anti-p73 (clone ER-15, Becton Dickinson). To identify deltaNp73, the monoclonal antibody anti-deltaNp73 (clone 38C674) was purchased from Active Motif (Rixensart, Belgium). Primary antibodies were labelled with goat anti-mouse IgG-FITC. To evaluate background staining, goat anti-mouse IgG-FITC was used.

N-myc was also explored by Western blot analysis using monoclonal antibodies against N-myc (1 : 250, clone NCM II 100; mouse IgG1). *β*-Actin (1 : 1.000, mouse; Sigma, Taufkirchen, Germany) served as the internal control.

To investigate N-myc coding mRNA, RT/PCR has been carried out as described above. The N-myc primer sequences were as follows: forward: 5′GACCACAAGGCCCTCAGTAC 3′; reverse: 5′GTGGATGGGAAGGCATCGTT 3′.

### Statistics

All experiments were performed 3–6 times. Statistical significance was investigated by the Wilcoxon–Mann–Whitney *U*-test. Differences were considered statistically significant at *P*<0.05.

## RESULTS

### VPA downregulates cell adhesion of CDDP- and VCR-resistant UKF-NB-3 tumour cells

**Figure 1:**
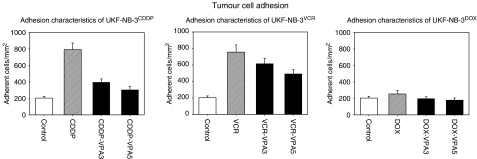
Valproic acid treatment causes adhesion blockade in CDDP- and VCR-resistant NB cells. The figure depicts adhesion capacity of parental UKF-NB-3 (control) *vs* VCR- (UKF-NB-3^VCR^), CDDP- (UKF-NB-3^CDDP^) or DOX-resistant NB subpopulations (UKF-NB-3^DOX^) *vs* resistant cell lines treated with 1 mM VPA for 3 (VPA3) or 5 days (VPA5). Neuroblastoma cells were added at a density of 0.5 × 10^6^ cells/well to HUVEC monolayers for 60 min. Non-adherent tumour cells were washed off in each sample, the remaining cells were fixed and counted in five different fields (5 × 0.25 mm^2^) using a phase contrast microscope. Adhesion capacity is depicted as tumour cell adhesion mm^−2^ (mean±s.d.; *n*=6).

Adhesion of UKF-NB-3, UKF-NB-3^CDDP^, UKF-NB-3^VCR^ or UKF-NB-3^DOX^ was quantified 60 min after plating the cells on to an endothelial cell monolayer (Figure 1). Nearly 200 parental (drug-sensitive) UKF-NB-3 cells mm^−2^ were attached to HUVEC during this time (SD_interassay_ <50%, SD_intraassay_ <10%). The amount of adherent cells increased fourfold when UKF-NB-3 became resistant to CDDP or VCR. Doxorubicin resistance did not induce any effects on tumour cell binding to HUVEC. Table 1 provides a profile of cross-resistance among the sublines examined.

The application of 1 mM VPA to UKF-NB-3^CDDP^ or UKF-NB-3^VCR^ significantly blocked the cellular adhesion process (Figure 1). A 5-day incubation period evoked stronger effects than a 3-day incubation period. Notably, treatment of UKF-NB-3^CDDP^ with VPA completely reverted the elevated adhesion behaviour induced by drug resistance. Valproic acid also acted on the parental cell lines, as evidenced by a significant downregulation of the number of adherent UKF-NB-3. The 60-min adhesion rate was reduced by 59.4±18.6% (*n*=6).

The PicoGreen assay did not reveal any proliferative activity during the experiment, which rules out the possibility that adhesion differences between drug resistant, VPA-treated and control NB cells may be caused by different cell growth capacity.

### VPA upregulates NCAM surface expression

**Figure 2:**
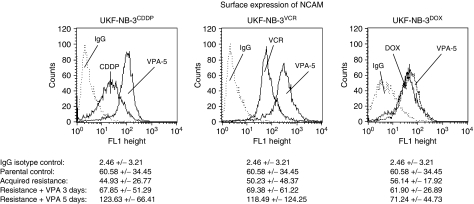
Valproic acid treatment enhances NCAM surface expression in CDDP- and VCR-resistant NB cells. Histograms plots show NCAM surface expression on untreated *vs* VPA-treated UKF-NB-3^CDDP^, UKF-NB-3^VCR^ and UKF-NB-3^DOX^. IgG isotype controls are also included. The complete experimental data set (MFU±s.d.; *n*=6) is given below the histograms. Tumour cells were disaggregated mechanically in each experiment and washed in blocking solution. An FITC-conjugated monoclonal antibody anti-CD56, clone 16.2, was used to detect the NCAM 120, 140 and 180 kDa isoform. A mouse IgG1-FITC served as the isotype control (IgG). Fluorescence was analysed using a FACScan flow cytometer, and a histogram plot (FL1-Height) was generated to show FITC fluorescence.

Figure 2 depicts one representative histogram analysis of NCAM receptor expression. The histogram presentation concentrates on isotype controls and NCAM-specific fluorescence of untreated *vs* VPA-treated (5-day treatment)-resistant tumour cells. Results of parental UKF-NB-3 and of resistant tumour cells treated for 3 days with VPA were not inserted, because the histograms overlapped, making the figures unclear. However, the complete experimental data set (MFU±s.d.; *n*=6) is given below the histogram.

Reduction of NCAM surface expression was observed on UKF-NB-3^CDDP^ and UKF-NB-3^VCR^, when compared to the parental control cell line. However, no changes were seen with NCAM expression on UKF-NB-3^DOX^, compared to the controls. Treatment of CDDP- or VCR-resistant cell lines with VPA led to a significant increase in NCAM, which exceeded the control values after a 5-day incubation period.

Valproic acid also acted on the parental cell lines, as evidenced by a significant upregulation of NCAM by +56.9±22.8% (*n*=5).

### VPA enhances NCAM protein content

**Figure 3:**
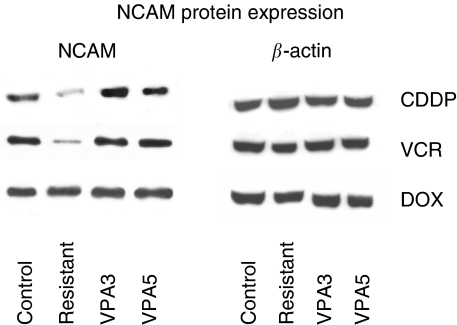
Western blot analysis of NCAM from the proteins of UKF-NB-3, UKF-NB-3^CDDP^ UKF-NB-3^VCR^ UKF-NB-3^DOX^ and resistant subpopulations treated with VPA for 3 (VPA3) or 5 days (VPA5). Cell lysates were subjected to SDS–PAGE and blotted on the membrane incubated with anti-NCAM (clone 16.2) monoclonal antibodies. *β*-Actin served as the internal control. The figure shows one representative from three separate experiments.

Similar to the modifications of the NCAM surface expression level, UKF-NB-3^CDDP^ and UKF-NB-3^VCR^ were characterised by a strong reduction of NCAM proteins when compared to the parental UKF-NB-3 control cells (Figure 3). No differences were seen between UKF-NB-3 and UKF-NB-3^DOX^.

When UKF-NB-3^CDDP^ or UKF-NB-3^VCR^ were treated with VPA for 3 or 5 days, NCAM protein content became upregulated, partially exceeding the control values. Applying VPA to UKF-NB-3^DOX^ did not induce any alterations in NCAM, independent of the incubation time.

### VPA modifies NCAM mRNA expression

**Figure 4:**
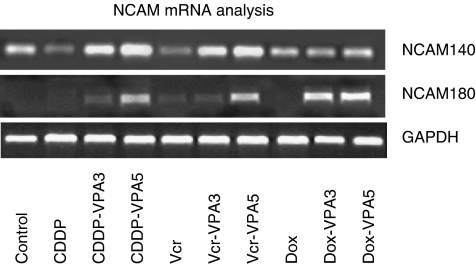
RT–PCR analysis of NCAM 140 and 180 kDa RNA in UKF-NB-3, UKF-NB-3^CDDP^ UKF-NB-3^VCR^ UKF-NB-3^DOX^ and resistant subpopulations treated with VPA for 3 (VPA3) or 5 days (VPA5). Ribonucleic acid were extracted, reverse-transcribed and submitted to semiquantitative RT–PCR using gene-specific primers as indicated in Materials and Methods. The internal control for the RT–PCR reaction was performed by running parallel reaction mixtures with the housekeeping gene GAPDH. The figure shows one representative from three separate experiments.

Assessment of NCAM mRNA showed distinct expression of mRNA encoding the 140 kDa isoform in UKF-NB-3 control cells, which however became down-modulated in UKF-NB-3^CDDP^ or UKF-NB-3^VCR^ (Figure 4). Only slight differences were visualised between UKF-NB-3 and UKF-NB-3^DOX^. The presence of VPA was accompanied by elevated 140 kDa mRNA levels in UKF-NB-3^CDDP^ or UKF-NB-3^VCR^. This effect was not seen in UKF-NB-3^DOX^, irrespective if VPA was applied for 3 or 5 days.

Messenger ribonucleic acid encoding the 180 kDa isoforms was not detected in parental UKF-NB-3, nor in UKF-NB-3^CDDP^, or UKF-NB-3^DOX^, and only very weakly expressed in UKF-NB-3^VCR^. Surprisingly, VPA evoked NCAM 180 kDa mRNA synthesis already after 3 days in UKF-NB-3^DOX^. The same phenomenon was observed after a 5-day VPA treatment in UKF-NB-3^CDDP^ or UKF-NB-3^VCR^.

### VPA alterates p73, deltaNp73 and N-myc expression

p73, deltaNp73 and N-myc have been identified to trigger NCAM expression (Blaheta *et al*, 2004). They were therefore used as biomarkers to further explore the influence of VPA on NCAM-triggered NB adhesion. p73 was detected in UKF-NB-3, expression of which was significantly reduced in UKF-NB-3^CDDP^ and UKF-NB-3^VCR^. Doxorubicin resistance was not accompanied by a distinct p73 downregulation (Table 2). When VPA was added to the cell cultures, downregulation of p73 seen in UKF-NB-3^CDDP^ and UKF-NB-3^VCR^ was reverted and the protein became upregulated after 3 and 5 days. deltaNp73 became enhanced in UKF-NB-3^CDDP^ and UKF-NB-3^VCR^ compared to the drug naïve UKF-NB-3 controls. Application of VPA led to a significant reduction of deltaNp73 in CDDP- or VCR-resistant cell lines.

**Figure 5:**
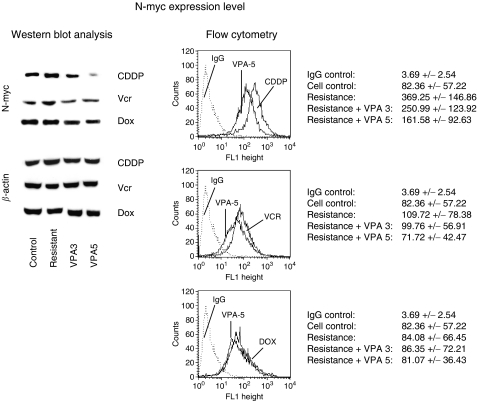
Valproic acid alters N-myc expression in CDDP- and VCR-resistant UKF-NB-3 cells. The right part of the figure depicts results from flow cytometry analysis. To allow intracellular staining, tumour cells were fixed and permeabilised by methanol–acetone before antibodies were added. To identify N-myc, the monoclonal anti-N-myc antibody clone NCM II 100 was used. Primary antibodies were labelled with goat anti-mouse IgG-FITC. Background staining was evaluated by goat anti-mouse IgG-FITC. The histograms plots show N-myc expression level in untreated *vs* VPA-treated UKF-NB-3^CDDP^, UKF-NB-3^VCR^ and UKF-NB-3^DOX^. IgG isotype controls are also included. The complete experimental data set (MFU±s.d.; *n*=6) is given alongside. The left part of the figure shows Western blot analysis of N-myc from the proteins of UKF-NB-3, UKF-NB-3^CDDP^ UKF-NB-3^VCR^ UKF-NB-3^DOX^ and resistant subpopulations treated with VPA for 3 (VPA3) or 5 days (VPA5). Cell lysates were subjected to SDS–PAGE and blotted on the membrane incubated with anti-N-myc monoclonal antibodies. *β*-Actin served as the internal control. The figure shows one representative from three separate experiments.

N-myc proteins were enhanced in UKF-NB-3^CDDP^ and UKF-NB-3^VCR^, but not in UKF-NB-3^DOX^, compared to the UKF-NB-3 control cell line (Figure 5). Moderate N-myc downregulation was induced by VPA in UKF-NB-3^VCR^. N-myc became nearly undetectable in UKF-NB-3^CDDP^ after a 5-day treatment with VPA. The distinct influence of VPA on UKF-NB-3^CDDP^ or UKF-NB-3^VCR^ was also proven by flow cytometry, which revealed left shifting of the N-myc-specific fluorescence intensity, indicating N-myc loss. N-myc histogram analysis concentrates on isotype controls and specific fluorescence of untreated *vs* VPA-treated (5-day treatment) UKF-NB-3^CDDP^, UKF-NB-3^VCR^ and UKF-NB-3^DOX^. The complete experimental data set (MFU±s.d.; *n*=6) is given below the histogram.

### VPA acts on further NB tumour cell lines

To strengthen the relevance of our findings, further NB tumour cell lines were included in the study. Analysis of UKF-NB-2 and drug-resistant sublines indicated a significant increase in UKF-NB-2^CDDP^, UKF-NB-2^VCR^ and UKF-NB-2^DOX^ cell adhesion, compared to the parental controls. Adhesion correlated inversely with the NCAM surface level (Table 3). A similar phenomenon was observed in CDDP- (moderate) and VCR-resistant UKF-NB-6 cell lines. A 3-day VPA treatment blocked cell binding to HUVEC in UKF-NB-2 and their drug-resistant sublines, and upregulated NCAM expression. Valproic acid also acted on UKF-NB-6, UKF-NB-6^CDDP^, UKF-NB-6^VCR^ and SKNSH. However, although VPA (partially) reverted the increased adhesion phenotype and restored NCAM levels, it did not re-sensitise the drug-resistant NB cells to VCR, DOX or CDDP (data not shown).

N-myc was significantly diminished by VPA in UKF-NB-2, UKF-NB-2^CDDP^, UKF-NB-6 and UKF-NB-6^CDDP^, compared to the controls. However, no differences were seen between VPA-treated and non-treated VCR or DOX-resistant tumour cell lines.

To better interprete our data, antiproliferative effects of VPA on the chemosensitive *vs* chemoresistant NB cells were analysed in final experiments. Table 4 document growth inhibition by this compound, whereas higher VPA concentrations were necessary to reach 50% reduction in chemotherapy-resistant cell lines compared to the parental controls.

## DISCUSSION

Based on this cell culture model, we have demonstrated that drug-resistant NB cancer cells develop an increased malignant phenotype as evidenced by enhanced adhesion to vascular endothelial cells, accompanied by significant downregulation of the adhesion receptor NCAM. Valproic acid reverted this process by downregulating cell adhesion and upregulating NCAM expression. Considering clinical utility, it is encouraging that VPA was active in NB cell lines resistant to existing chemotherapies, since overcoming resistance to anticancer agents is a major challenge in the development of novel antitumour protocols.

In fact, several cases have been documented showing antineoplastic effects of VPA in patients with relapsed tumours. When VPA was given as maintenance therapy for childhood malignant glioma after postoperative combined chemotherapy and irradiation, about 10% of these patients were maintained in continuous complete remission and an equal number of patients showed at least partial responses (Blaheta *et al*, 2005). An additional pediatric patient with glioblastoma multiforme responded to VPA after showing progressive disease shortly after having received combined chemotherapy and irradiation as well as topotecane (Witt *et al*, 2004). Another pediatric patient suffering from a relapsed supratentorial primitive neuroectodermal tumour while receiving chemotherapy (CCNU, VCR and cisplatinum) after total resection and irradiation showed conspicuous signs of glial differentiation induction and a non-malignant morphology on histological examination. This patient had received VPA for epilepsy treatment for a period of several months before the tumour recurred (Driever *et al*, 2004).

*In vitro*, VPA has been shown to inhibit proliferation in acute myeloid leukaemia cells expressing P-glycoprotein (P-gp) and MDR-associated protein 1 (Tang *et al*, 2004), and to increase sensitivity towards apoptosis in hepatoma cells resistant to epirubicin (Schuchmann *et al*, 2006). Although the underlying mode of action has not been explored in these studies, the data clearly indicate that VPA may alter the malignant behaviour of tumours that do not respond to chemotherapy. With particular emphasis on NB, VPA significantly prevented the interaction between tumour cells and endothelium. This finding is important, because binding of single cancer cells to the vessel wall represents the first step in the haematogenous invasion cascade proceeding transendothelial migration and invasion into surrounding tissue. We therefore conclude that VPA may have a direct impact on metastasis formation. In good accordance to this hypothesis, VPA enhanced the NCAM surface level, expression of which is strongly involved in tumour cell adhesion and penetration.

In primitive neuroectodermal tumour cells, an increase in NCAM was paralleled by a significant reduction in cellular motility and adhesion capacity (Owens *et al*, 1998; Prag *et al*, 2002). In a rat model, NCAM-transfected glioma tumour cells became less invasive and destructive than control cells with a low NCAM expression level (Edvardsen *et al*, 1994). Diminished expression of NCAM was also associated with clinically aggressive colon cancers (Sampson-Johannes *et al*, 1996; Roesler *et al*, 1997; Huerta *et al*, 2001), and dissemination of pancreatic *β*-tumour cells (Perl *et al*, 1999; Cavallaro *et al*, 2001). Tezel *et al* (2001) suggested that NCAM expression in tubular adenocarcinoma of the pancreas has a significant impact on overall patient survival. We recently demonstrated an inverse correlation between NCAM expression and NB cell adhesion, assessed on 11 NB cell lines. In particular, transfection with a cDNA encoding the human NCAM-140 kD isoform enhanced NCAM expression and diminished initial NB cell adhesion, treatment with NCAM antisense oligonucleotides reduced NCAM surface level and induced upregulation of NB cell adhesion to endothelium (Blaheta *et al*, 2002). It is currently assumed that NCAM, in its function as a homophilic receptor, stabilises the primary tumour or tumour cell aggregates, while circulating in the blood vessels. Reduction of the NCAM expression level might lead to a reduction in cell–cell binding forces, and hence to the release of tumours as single cells. The less NCAM, the more metastatic cells leave the tumour mass, and the more penetration events can take place (Blaheta *et al*, 2002). Consequently, NCAM upregulation observed in CDDP- and VCR-resistant NB tumour cells under VPA might reduce cell transmigration and extravasation processes.

There is some evidence from the literature that N-myc downregulates NCAM expression, thus increasing the invasiveness of NB cells. Transfection of the rat NB cell line B104 with an N-myc expression vector resulted in a dramatic reduction in the levels of NCAM polypeptides and mRNAs, and increased metastatic ability (Akeson and Bernards, 1990). Cytomegalovirus-induced acceleration of NB adhesion and transendothelial penetration was evoked by increasing N-myc protein content, accompanied by a diminished NCAM surface level (Blaheta *et al*, 2004). The present data reveal strong upregulation of N-myc in UKF-NB-3^CDDP^, compared to the parental cells, process of which was coupled to NCAM loss and enhanced adhesion capacity. It may therefore be concluded that N-myc plays an important role in NCAM-driven NB adhesion, and that VPA has an impact on N-myc protein expression. Nevertheless, the situation is more complex than initially thought. Our analysis on further NB cell lines indicated that VPA reverts cell adhesion, restored NCAM and suppressed N-myc expression level on UKF-NB-2, UKF-NB-6 and their CDDP-resistant sublines. However, VPA effects on VCR-resistant sublines were not accompanied by N-myc alterations. Furthermore, incubation of multidrug-resistant SKNSH with VPA induced very strong adhesion blockade and NCAM upregulation, although N-myc was detected in non-treated controls just very slightly over threshold values. Based on this, we assume that endogenous N-myc expression level of NB cells may not correlate with their responsiveness to VPA-induced NCAM upregulation, and VPA-induced loss of N-myc may be limited to CDDP-resistant and drug-sensitive tumour cells.

p73 and deltaNp73, an isoform of p73 lacking the N-terminal transactivation domain, are both suggested to be associated with NCAM expression. *In vitro* experiments demonstrated that transfected full-length p73 cDNA induces expression of NCAM and downregulation of N-myc in N1E-115 NB cells. Inversely, transfection of dominant-negative p73 abrogated the transactivation of the NCAM promoter (De Laurenzi *et al*, 2000). Based on UKF-NB-4 tumour cells, we have recently postulated a direct association between tumour progression, upregulation of N-myc and deltaNp73, and downregulation of p73 and NCAM (Blaheta *et al*, 2004). According to this statement, VPA evoked UKF-NB-3 adhesion blockade was accompanied by diminished N-myc and deltaNp73, and an enhanced p73 and NCAM level. With special respect to the UKF-NB-3 model, VPA may interfere in the N-myc/deltaNp73 signalling system that causes, as at least one consequence, distinct upregulation of NCAM biosynthesis and receptor processing. Neural cell adhesion molecule processing finally attributes to the lowered invasive capacity of the tumour cells.

To summarise, evidence is presented showing that drug-resistant NB cells are sensitive to VPA treatment. Valproic acid distinctly reduced the invasive properties of NB and may therefore be well suited to amend the current treatment protocol with particular emphasis on those tumours that do not respond to chemotherapy. Nevertheless, our data are particularly limited to four NB cell lines. Therefore, the hypothetical possibility that VPA might overcome drug resistance in general needs further investigation. Remarkably, VPA blocked cell adhesion of parental, but not of DOX-resistant UKF-NB-3 or UKF-NB-6 tumour cells in our assay. This implies that DOX may, under certain circumstances, induce resistance to VPA. A similar phenomenon was found using NB cells with ‘naturally’ arised DOX resistance, SMS-KANR and SMS-KCNR (Reynolds *et al*, 1986). Tabe *et al* (2006) demonstrated in this context that the HDAC-inhibitor FK228 induces P-gp expression and prevents growth inhibition and apoptosis in acute promyelocytic leukaemia cells subsequently incubated with DOX. Okada *et al* (2006) observed resistance development in DOX-resistant clones of osteosarcoma and Ewing's family of tumours after exposure to FK228. Nevertheless, FK228 is chemically different from VPA and, therefore, further experiments are necessary to explore this issue.

Differences between VCR/CDDP- and DOX-resistant cell lines may also point to different resistance mechanisms that are operational in these cell lines. Suppression of MAP kinase (MEK-ERK) signalling has been observed in NB cells with acquired resistance to DOX (Mattingly *et al*, 2001; Armstrong *et al*, 2006). Down-modulation of ERK1/2 phosphorylation has further been documented in NB cells with acquired resistance to CDDP or VCR. However, these cell lines were additionally characterised by a distinct Akt activation (Kotchetkov *et al*, 2005; Servidei *et al*, 2006). Therefore, although purely speculative, fine-tuned alterations of the ERK and Akt signalling system may be – at least partially – responsible for establishing VCR and CDDP, but not DOX resistance in our NB cell model. However, detailed knowledge of resistance mechanism in individual cancer cells is necessary to allow better prediction of the clinical use of VPA.

Other HDAC inhibitors have also been shown to inhibit tumour growth and to overcome multidrug resistance. Notably, suberoylanilide hydroxamic acid (SAHA) has been demonstrated to act on VCR-resistant leukaemia cell lines (Ruefli *et al*, 2002), adriamycin-resistant breast (Castro-Galache *et al*, 2003) and paclitaxel-resistant ovarian cancer cells (Sonnemann *et al*, 2006). SAHA at 5 *μ*M significantly diminished UKF-NB-3^CDDP^ or UKF-NB-3^VCR^ proliferation and cell adhesion to HUVEC in our own experiments (data not shown). Histone deacetylase inhibitors different from VPA may therefore be considered to become additional options for the treatment of drug-resistant NB. However, detailed studies are necessary to explore their clinical value.

## Figures and Tables

**Table 1:** Level of drug resistance, indicated as IC_50_ values (ng ml^−1^)

**Cell line**	**IC_50_VCR**	**IC_50_ DOX**	**IC_50_ CDDP**
UKF-NB-2	0.74±0.15	8.2±2.5	136±41
UKF-NB-2^VCR^	47.9±11.2	114.6±20.7	154±32
UKF-NB-2^DOX^	23.4±6.4	40.7±12.3	225±38
UKF-NB-2^CDDP^	1.35±0.47	17.4±7.7	819±52
UKF-NB-3	0.35±0.09	2.25±0.78	188±22
UKF-NB-3^VCR^	52.7±9.8	69.1±15.8	532±68
UKF-NB-3^DOX^	205±51.2	62.5±15.4	231±22
UKF-NB-3^CDDP^	0.84±0.31	17.3±2.4	1278±177
UKF-NB-6	1.91±0.18	3.5±0.9	114±22
UKF-NB-6^VCR^	57.2±11.2	108±12	523±69
UKF-NB-6^DOX^	19.5±3.4	17.2±6.5	102±18
UKF-NB-6^CDDP^	3.72±0.76	21.4±4.5	2102±150
SKNSH	7.9±1.3	25.4±10.1	47.1±13.5

Values are from six independent experiments±s.d.

**Table 2:** Mean fluorescence units showing influence of VPA on p73 and deltaNp73 expression in drug-resistant neuroblastoma cells

**Cell line**	**p73**	**deltaNp73**
UKF-NB-3	18.29±6.25	11.51±8.34
CDDP	3.38±3.64^a^	19.71±7.68^a^
CDDP+VPA3	8.16±5.66^b^	12.44±6.94^b^
CDDP+VPA5	12.8±7.32^b^	11.06±6.72^b^
VCR	5.23±4.78^a^	26.17±13.95^a^
VCR+VPA3	13.78±6.13^b^	17.33±8.99^b^
VCR+VPA5	17.62±8.72^b^	11.23±6.85^b^
DOX	15.46±8.29	10.02±4.29
DOX+VPA3	17.90±11.21	10.64±6.78
DOX+VPA5	19.95±13.96	11.95±3.81

Values are from six independent experiments±s.d. CDDP indicates UKF-NB-3^CDDP^, VCR indicates UKF-NB-3^VCR^, DOX indicates UKF-NB-3^DOX^. VPA was added for 3 (VPA3) or 5 days (VPA5).

a Indicates significant difference to UKF-NB-3.

b Indicates significant difference to the drug-resistant NB sublines.

**Table 3:** Comparative analysis of adhesion capacity, NCAM and N-myc expression level of several neuroblastoma cells and drug-resistant sublines

**Cell line**	**Cell adhesion to HUVEC (cells mm^−2^)**	**NCAM expression (MFU)**	**N-myc expression (MFU)**
UKF-NB-2	75.2	188.2	40.1
UKF-NB-2±VPA	60.8^c^	212.6^c^	28.6^c^
UKF-NB-2^CDDP^	318.4^a^	82.8^a^	39.6
UKF-NB-2^CDDP^±VPA	175.2^b^	168.9^b^	32.2^b^
UKF-NB-2^VCR^	245.6^a^	99.7^a^	44.7
UKF-NB-2^VCR^±VPA	121.6^b^	154.6^b^	42.3
UKF-NB-2^DOX^	172.6^a^	127.9^a^	38.8
UKF-NB-2^DOX^±VPA	124.5	143.5	39.4
UKF-NB-6	72.4	256.8	339.3
UKF-NB-6±VPA	41.0^c^	316.8^c^	238.0^c^
UKF-NB-6^CDDP^	81.6	248.7	305.8
UKF-NB-6^CDDP^±VPA	56.8^b^	283.8^b^	224.2^b^
UKF-NB-6^VCR^	115.6^a^	181.0^a^	298.9
UKF-NB-6^VCR^±VPA	79.2^b^	284.2^b^	331.6
UKF-NB-6^DOX^	73.8	251.3	344.0
UKF-NB-6^DOX^±VPA	71.9	254.5	321.7
SKNSH	56.8	318.9	17.7
SKNSH±VPA	27.2^c^	812.1^c^	18.6

MFU=mean fluorescence units.

a Indicates significant difference to the parental neuroblastoma cell line.

b Indicates significant difference to the drug-resistant tumour sublines.

c Indicates significant difference between VPA-treated parental cells and their non-treated controls. Mean standard deviations were as follows: cell adhesion_intra-assay_<25%, cell adhesion_inter-assay_<80%. NCAM expression_intra-assay_<5%, NCAM expression_inter-assay_<30%. N-myc expression_intra-assay_<5%, N-myc expression_inter-assay_<50%.

**Table 4:** Antiproliferative effects of VPA, indicated as IC_50_ values (mM)

**Cell line**	**IC_50_**
UKF-NB-2	0.44±0.31
UKF-NB-2^CDDP^	1.85±0.58
UKF-NB-2^VCR^	1.36±0.62
UKF-NB-2^DOX^	1.44±0.42
UKF-NB-3	1.03±0.37
UKF-NB-3^CDDP^	1.51±0.44
UKF-NB-3^VCR^	1.88±0.57
UKF-NB-3^DOX^	2.18±0.76
UKF-NB-6	1.27±0.53
UKF-NB-6^CDDP^	1.53±0.46
UKF-NB-6^VCR^	2.36±0.71
UKF-NB-6^DOX^	0.93±0.39
SKNSH	3.16±0.78

Values are from six independent experiments±s.d.
